# The role of valve stiffness in the insurgence of deep vein thrombosis

**DOI:** 10.1038/s43246-020-00066-2

**Published:** 2020-09-16

**Authors:** Zoe Schofield, Hosam Alden Baksamawi, Joana Campos, Alessio Alexiadis, Gerard B. Nash, Alexander Brill, Daniele Vigolo

**Affiliations:** 1 School of Chemical Engineering, University of Birmingham, Birmingham, B15 2TT UK; 2 Physical Sciences for Health, University of Birmingham, Birmingham, B15 2TT UK; 3 Institute of Cardiovascular Sciences, University of Birmingham, Birmingham, B15 2TT UK; 4 Department of Pathophysiology, Sechenov First Moscow State Medical University (Sechenov University), Moscow, Russia; 5 Centre of Membrane Proteins and Receptors, University of Birmingham and Nottingham, The Midlands, UK

**Keywords:** Thrombosis, Biomedical engineering, Fluid dynamics, Thrombosis, Bioinspired materials

## Abstract

Deep vein thrombosis is a life-threatening development of blood clots in deep veins. Immobility and blood flow stagnancy are typical risk factors indicating that fluid dynamics play an important role in the initiation of venous clots. However, the roles of physical parameters of the valves and flow conditions in deep vein thrombosis initiation have not been fully understood. Here, we describe a microfluidics in vitro method that enabled us to explore the role of valve elasticity using in situ fabrication and characterisation. In our experimental model the stiffness of each valve leaflet can be controlled independently, and various flow conditions were tested. The resulting complex flow patterns were detected using ghost particle velocimetry and linked to localised thrombus formation using whole blood and an aqueous suspension of polystyrene particles. In particular, valves with leaflets of similar stiffness had clot formation on the valve tips whereas valves with leaflets of different stiffness had clot formation in the valve pocket.

## Abstract (web summary)

Deep vein thrombosis is the clotting of blood in deep veins. Here, a microfluidic device containing flexible valves fabricated in-situ is used to investigate the effects of blood flow conditions and valve elasticity on thrombus formation, revealing the circumstance under which clotting occurs.

## Introduction

Deep vein thrombosis (DVT) is a disease in which blood clots form in the deep veins usually, but not exclusively, of the legs. These clots can become unstable, get detached and travel to the lungs causing a potentially fatal condition known as pulmonary embolism (PE)^1^. DVT and PE, designated together as venous thromboembolism (VTE), develop in about 900,000 Americans annually leading to high morbidity and mortality^2^. Despite this, the mechanisms underlying DVT initiation remain unclear.

It is a general assumption that DVT starts in the venous valve sinus due to a stagnant region behind the leaflet^3^. In contrast to arterial thrombosis that usually results from atherosclerotic plaque rupture and platelet accrual at the exposed subendothelial matrix^4^, thrombosis in veins is not accompanied by endothelial denudation^5^. Blood flow stagnation up to stasis is considered one of the major factors triggering DVT^6^. Interrupted flow usually results from immobilisation of the person for reasons such as bed-ridden position after surgery, paralysis or long-haul flights. For example, in paediatric patients, lack of movement exceeding 3 days is a recognised risk factor for venous thrombosis with each additional day of stay at hospital increasing the probability for DVT by 3%^7^. Blood flow in veins becomes slower^8^ and valves stiffer with age which correlates with greatly increased prevalence of DVT in elderly people^9^. Slow blood flow causes low oxygen tension (hypoxia) in the venous wall, which initiates inflammation-like processes leading eventually to thrombosis^10^.

Thus, the significance of reduction in flow as a risk factor implies that local characteristics of flow play a leading role in DVT pathogenesis.

In vivo models of DVT include flow restriction in a major vein (usually the inferior vena cava, IVC) in rodents to induce thrombus formation^11–13^. These models have been useful for the development, for example, of drugs to treat DVT^12,14^, however, they do not allow for exploring the importance of physical parameters triggering thrombosis (blood flow, elasticity of the valve, etc.). Indeed, a specific pattern of flow inside valves has been demonstrated. Two counter-rotating vortices are formed in the valve space contributing to the prolonged residence of blood in the area^6^. In addition to blood stasis in the valve pocket, there is mixing at the valve tips due to vortex formation. The vortex encourages circulation of the cellular and humoral prothrombotic factors facilitating their interaction.

Despite their role in venous thrombosis, flow patterns around the valves have been scarcely investigated in part due to lack of an appropriate in vitro model that would recapitulate valves in a real vein. More recently, numerical simulations have paved the way for valve models^15–18^. However, there is a distinct lack of experimental validation.

Venous flow varies, dependent on factors, such as posture, muscular exertion, hydration, and vessel diameter^19^. Moreover, the local flow pattern is also affected by the geometry of the valves, and their mechanical properties. Definition of the roles of these mechanical parameters is paramount for understanding the mechanisms of DVT initiation. Therefore, micro-/milli-fluidics provides an ideal platform, allowing for highly controlled environments in which the size of the channels and the flow rates can be controlled precisely to mimic in vivo conditions^20–23^. For example, in the work by Lehmann et al.^24^, a series of rigid constructions mimicking a valve are used to study flow pattern changes at a fixed flow rate. In concert with our findings, this group observed two vortices forming in the valvular pocket. This work also showed the dependence of thrombus initiation on platelet accumulation and activation. However, rigid valves and a continuous flow may not reflect properly the sequence of events in real veins.

**Fig. 1:**
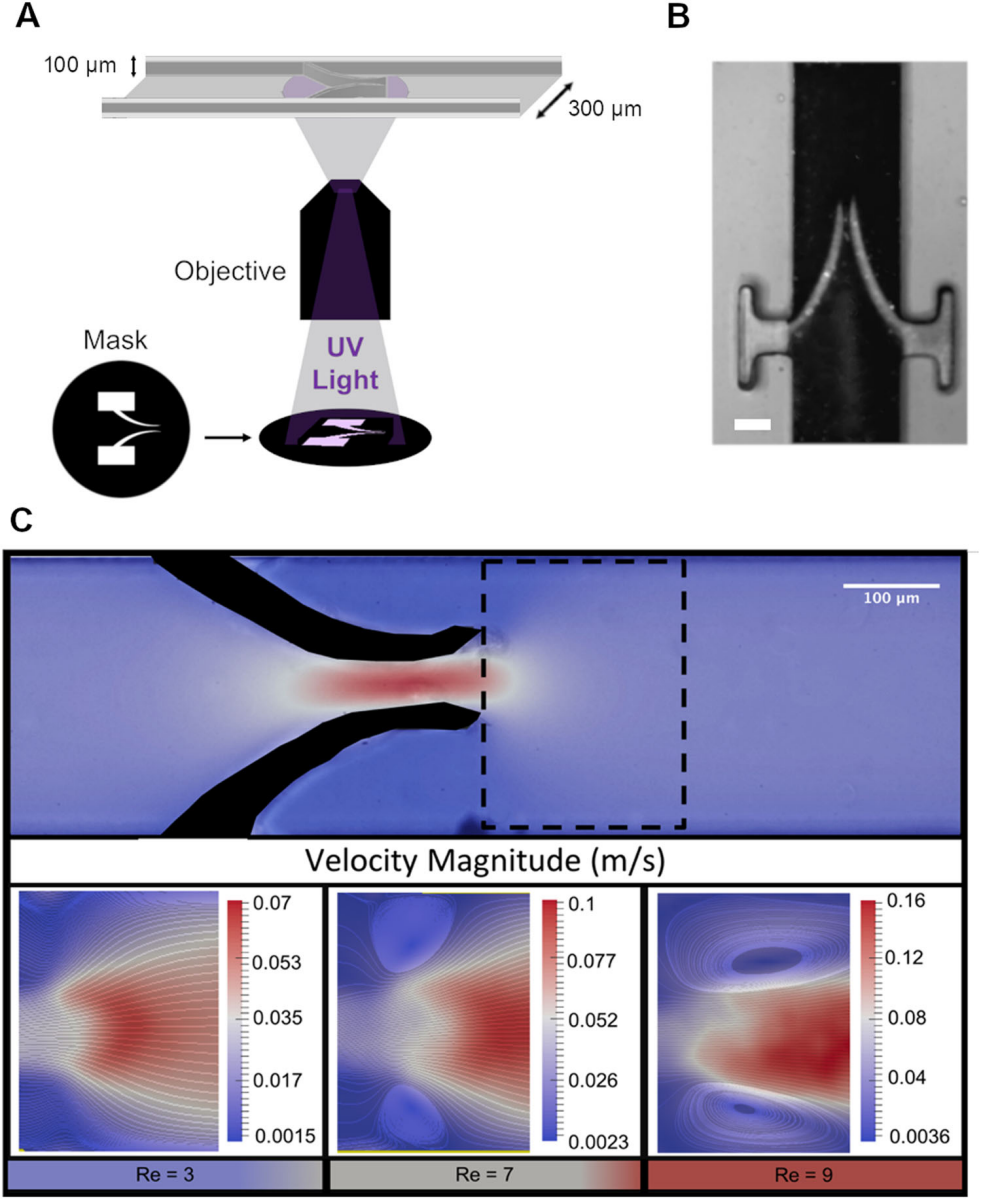
Vortex formation in the presence of flexible valves. **a** A Schematic diagram of mask alignment through the fluorescent light of an inverted microscope. The mask was designed in order to produce the desired valve size through a 20× objective. **b** Optical image of the flexible valve obtained. The length of the valve is about 330 µm, the width of each leaflet starts at about 45 µm and it tapers towards the tip. The initial gap in between the leaflets when there is no flow is about 30 µm. The scale bar represents 100 µm. **c** The top image is a velocity profile map with the site of vortex formation at the tip of the valve designated by the dashed line. Images below (from left to right) represent the vortex getting larger with increased flow rate (valve properties: 2% w/v [PI] and 40% w/v [PEGDA]).

Therefore, to precisely delineate the role of flow geometry in cell accumulation we exploited microfluidics to develop our in vitro model of a vein by creating a microchannel (typical cross section of 300 µm by 100 µm and several centimetres long as shown in Fig. 1a) made of polydimethylsiloxane (PDMS) using standard soft-lithography techniques^25^. We then incorporated flexible valves made of polyethylene glycol di-acrylate (PEGDA), by in situ photo-polymerisation (see Fig. 1a, b). Finally, we controlled the flow rate by a syringe pump (for continuous flow) or a pressure controller (for pulsed flow). This gave us the ability to mimic the motion of blood, both forward and backwards, in the presence of valves and demonstrated that variation in leaflet rigidity modified the flow patterns and induced the formation of aggregates of synthetic particles dispersed in water and platelets in whole blood. We thus revealed that (1) leaflet ends are primary points of initial accumulation, (2) increase in the rigidity of leaflets leads to higher particle accrual when rigidity of both leaflets in a valve is the same and (3) when rigidity of leaflets in a valve is different, particles accumulate preferably in the leaflet pocket as a result of the asymmetrical flow. An important feature of our model is the use of pulsatile flow, which mimics the effect of the muscle pump, accelerating blood flow in the veins. Thus, we demonstrate herein that changes in valve stiffness may affect thrombus formation (although stiffness of the vessel wall itself could not be taken into account in this model). This approach may be useful for future studies of the physical parameters predisposing to DVT initiation.

## Results and discussion

### Characterisation of flow pattern

In order to evaluate the changes in flow characteristics due to the presence of flexible valves, we used a recently developed all-optics technique, ghost particle velocimetry (GPV)^26–30^. GPV uses nanoparticles and bright field microscopy to generate a speckle pattern used to track the flow (see Supplementary Methods; Supplementary Figs. 1–3; Supplementary Movies 1 and 2).

Using GPV we were able to obtain detailed flow profiles. Venous flow is highly variable hence a range of flow rates (1–900 μL/min) and elasticity of valves (Young’s modulus, *E*, in the range 47–100 kPa) have been explored. To maintain hydrodynamic similarity between our in vitro and the in vivo condition we kept the Reynolds number (Re) in a physiological range typical for veins^31,32^. To model a ‘healthy’ vein, we used a flexible valve (*E* = 46.7 ± 5.2 kPa). This valve opens to its maximum extent at the highest flow rate emptying the stagnant pocket behind the valve. On the other hand, with a stiffer valve, an increased stagnant region and, consequently, a higher residence time of fluid behind the valve is detected. This is representative of elderly people where the valve tissue becomes less flexible^33^ due to an increase of fibrotic tissue^34^. In all cases we kept the stiffness of the vessel constant to emphasise the effect due solely to the valve elasticity.

Venous flow in general is not continuous; the blood is pushed back to the heart via muscle contractions (muscle pump) and back flow is prevented by the presence of valves^3^. These conditions were replicated using a pressure controller to pulse the flow through the valves causing them to open and close to better mimic the physiological conditions. In the presence of back flow, the valve closed and the flow recirculated in the valve pockets, encouraging vortex formation. The opening and closing of the valve permitted partial removal of the fluid trapped in the valve pocket proportionally to the valve stiffness.

### Vortex characterisation

As the stiffness of the valve increased, a larger space between the valve and the channel wall formed for a vortex to occur. The flow velocity between the valve leaflets became increasingly higher than the velocity through the main channel resulting in the formation of a jet responsible for the onset of a pair of symmetrical vortices, one at each leaflet tip (Fig. 1c and Supplementary Movie 1). These vortices increased the residence time of the fluid at the valve tips. An extended residence time in vivo can increase the likelihood of a thrombus forming due to a higher chance for activated cells to collide and form aggregates^35^.

In Fig. 1c, we show the velocity profile maps and streamlines collected using GPV. It is possible to notice a vortex forming at the valve tips at higher flow rates, identified by the Reynolds number, Re, defined as ${\rm{Re}} = \rho vD/\mu$, where *ρ* is the density, *v* the flow velocity, *D* the hydraulic diameter and *µ* the viscosity of the aqueous suspension.

**Fig. 2:**
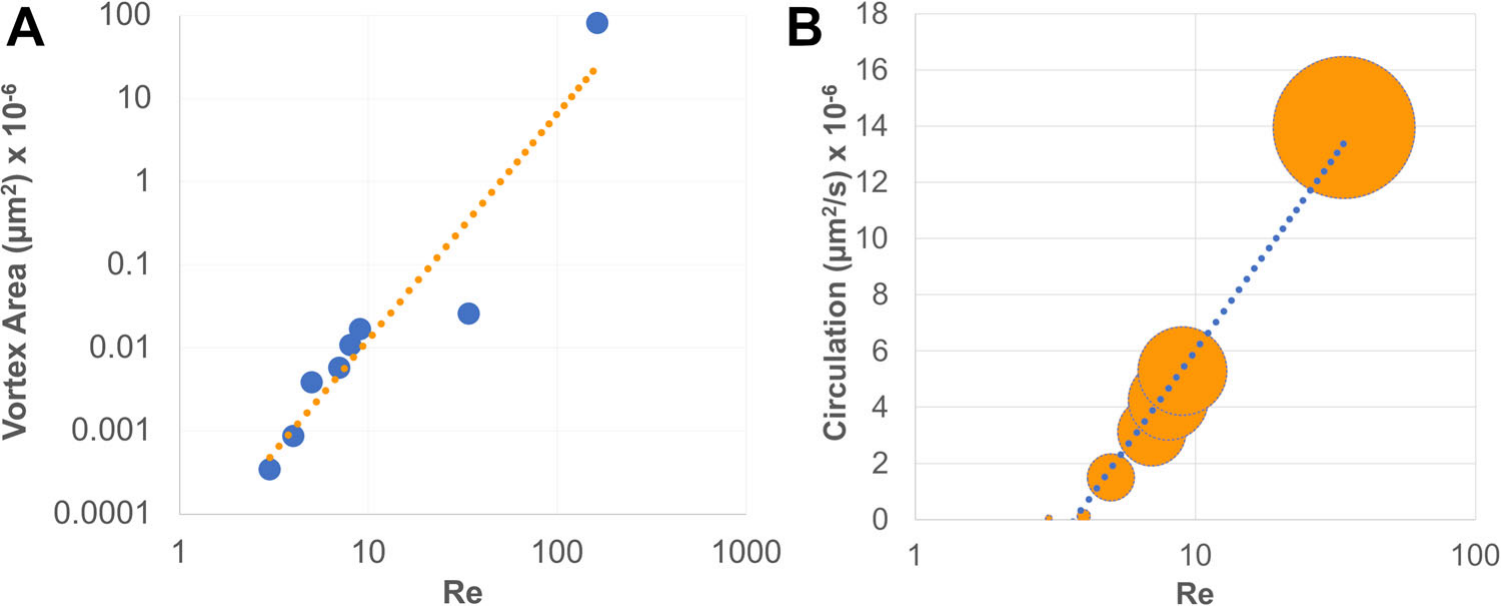
Vortex characteristics. **a** Vortex area increases as ≈Re^2.7^ (dashed line, *R*^2^ = 0.9057). **b** Circulation correlates with Re; the size of the marker point represents the minimum and maximum circulation calculated for the vortex developing at the tip of the valve and the dotted line represents a logarithmic fit (*R*^2^ = 0.9788).

Next, we demonstrated how the size of the vortices depended on the velocity of the fluid and the elasticity of the valve. Figure 2a shows the dependence of the vortex area on the Re which increases following a power law. Higher flow velocities contribute to the elongation of the vortex along the channel. As a consequence, also the circulation, defined as the integral of the tangential velocity around the vortex, increased with Re. Circulation varied within a vortex and reached a maximum at the boundary. We illustrated this by showing the minimal and maximal circulation values for each Re in Fig. 2b. Due to confinement within the microfluidic channel, the circulation tended to reach a plateau for high Re following a logarithmic behaviour (Fig. 2b), while it increases linearly with the vortex area (see Supplementary Fig. 4).

**Fig. 3:**
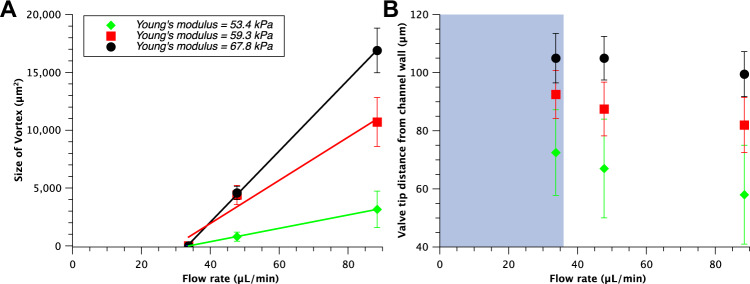
Vortex size dependence on flow rate for three different stiffnesses of the valve. **a** The size of the vortex increases linearly as the flow rate and/or the stiffness increases. The lines are linear fits. **b** Dependence of the degree of valve opening on flow rate for three different valve stiffnesses. Here, the smaller the distance from the channel wall the more the valve is open. As a consequence, for more flexible valves (lower Young’s modulus) the distance from the channel wall is smaller for a fixed flow rate compared to more rigid valve and the vortex associated is smaller (see panel **a**). In the plot, the grey area represents the region where no vortices were observed, independently of the stiffness of the valve tested. The error bars represent standard error.

In the presence of softer valves, higher deformation occurred that induced a reduction in vortex size (Fig. 3a). This is a consequence of the fact that these valves opened wider at the same flow rate compared to stiffer valves (Fig. 3b). This, in turn, generated a jet with lower flow velocity and thus a smaller vortex characterised by a lower tangent velocity and thus lower circulation. For the lowest flow rate used in our experiments, the vortex did not occur (Fig. 3b and Supplementary Movie 2), even for the highest Young’s modulus we tested. Thus, independently from the valve stiffness, we identified a threshold flow rate for which we did not observe the formation of vortices. This shows a clear link between the elasticity of the valve, flow velocity and vortex formation and identify the stiffness of the valve as the most significant parameter affecting the range of movements of the valve and therefore the size of the stagnant region^34,36^. In particular, at low flow rate typical of DVT, the position of the valve leaflets is solely determined by the stiffness of the valve and its dependence on the flow rate is negligible (Fig. 3b).

To further extend the range of Re investigated, a larger 3D printed device was used. This allowed us to study Re up to 163 (Fig. 2a) and evaluate the flow pattern by GPV. Although typical Re for healthy human veins can be higher^32^ we focused on the conditions favouring DVT, which is characterised by low blood flow rate or stagnation.

### In vitro thrombus formation

A general assumption is that venous thrombosis occurs in the valve pockets due to the stasis in the valvular pockets^37^. Yet it remains unclear where a thrombus is initiated. In order to investigate this experimentally we flowed an aqueous dispersion of polystyrene (PS) particles within the microfluidic device using either continuous or pulsed flow. PS particles have a tendency of forming aggregates due to Van der Waals and electrostatic interactions between themselves and solid surfaces. In addition, we performed similar experiments using whole blood with fluorescently labelled platelets from three different donors. In order to quantify the particle or platelets accrual on the valves, the surface area of the particle build up was evaluated using ImageJ^38^ (additional details available in the Supplementary Methods).

Using this approach, we studied the influence of the valve stiffness on the formation of aggregates. A healthy valve is flexible enough to permit an efficient exchange of blood with minimal flow stagnation; due to diseases or age valves tend to become stiffer. In addition, it is reasonable to speculate that the stiffness may change to the same or different extent for each leaflet^39^. Hence, we investigated particle agglomeration in valves with leaflets of identical (symmetrical valve) or different stiffness (asymmetrical valve).

### Symmetrical valve

Firstly, we tested continuous flow conditions with 200 nm PS particles. As we increased the flow rate, the particles build up decreased. In a typical experiment we monitored the particles accumulation by acquiring images every 100 ms for 5 min and evaluating the surface area covered by the aggregate to capture gradual build-up of particles over time. For example, at a flow rate of 50 μL/min, after 5 min, we detected a large accumulation of particles occupying a surface area of 10,500 ± 1300 μm^2^ on the valve tips, considerably larger than the accumulation at a flow rate of 150 μL/min (1300 ± 250 μm^2^) after the same time (a snapshot is shown in Supplementary Fig. 5). These results are consistent with DVT cases as there is an increased likelihood of developing DVT when immobile (long haul flights/surgery/bed ridden).

Next, the pulsed flow was tested. We used a pressure controller set to pulse between 0 and 100 mbar with a frequency of 1 Hz to flow an aqueous suspension of 3 and 10 µm diameter PS particles. These sizes were chosen as similar to platelets^40^ and red blood cells^41^. Maximal flow velocity through the channel was 0.2 m/s.

**Fig. 4:**
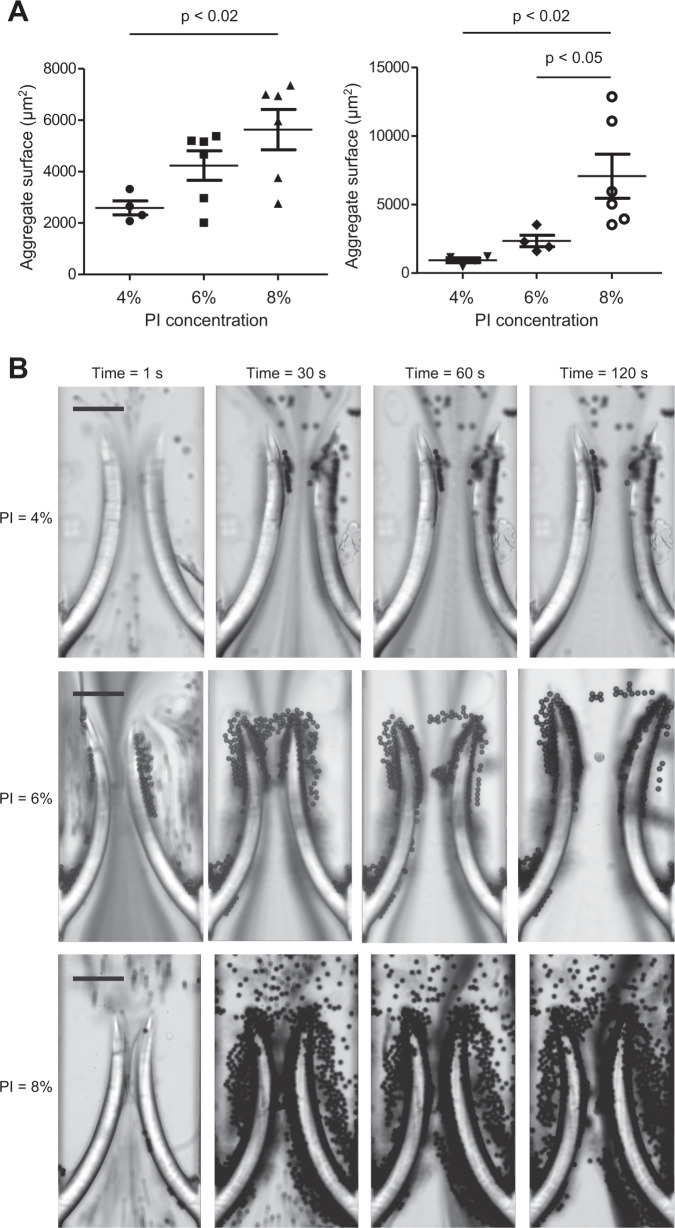
Accumulation behaviour for valves of different stiffness. **a** Positive correlation between valve stiffness and size of the aggregate formed by the accumulation of polystyrene particles of 3 µm (left, 0.1% w/v) and 10 µm (right, 1% w/v) in diameter in the case of symmetrical valve rigidity and pulsed flow condition (0–100 mbar, 1 Hz). The valves were made of 50% [PEGDA] and, 4, 6, or 8% [PI], respectively from softer to stiffer. The data were analysed statistically using one-way ANOVA followed by Tukey post hoc test. **b** Typical accumulation behaviour over time for 10 µm polystyrene particles (1% w/v) in diameter in the case of symmetrical valve rigidity and pulsed flow condition (0–100 mbar, 1 Hz). The scale bars represent 100 µm.

Movement of the valves caused the particles to build up at the tip of the leaflets, similarly to the continuous flow. By varying the elasticity of the valve, we found a direct correlation between stiffness and particle accumulation (Fig. 4b).

**Fig. 5:**
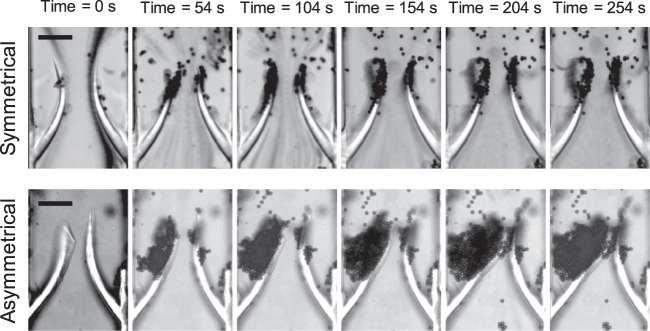
Accumulation behaviour for symmetrical and asymmetrical valves. Time-lapse images of particles building up over time on symmetrical (top) and asymmetrical (bottom) valves; both are 4% (w/v) PI and 50% (w/v) PEGDA but the right-hand side leaflet on the asymmetrical valve was exposed to UV light for a longer time compared to the left-hand side leaflet (500 and 300 ms, respectively) resulting in an asymmetrical stiffness. The scale bars represent 100 µm.

Particle build up was consistently localised at the valve tips (Figs. 4, 5, and Supplementary Movie 3).

**Fig. 6:**
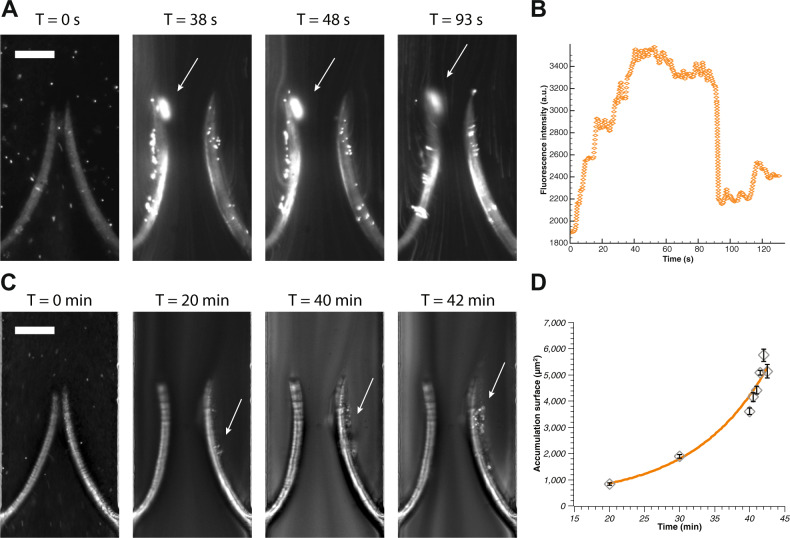
Platelets accumulation for symmetrical and asymmetrical valves. **a** Time-lapse images of blood aggregate building up over time on stiff symmetrical valves (see also Supplementary Movie 5). The aggregate forms rapidly within 30–40 s of pulsed flow conditions as shown by the fluorescently labelled platelets accumulating at the tip of the leaflets. It then increases slowly until eventually it detaches (in this case after 93 s). The arrow indicates the aggregate and the scale bar represents 100 µm. **b** Growth of the aggregate over time showing initial fast growth followed by a plateau and eventually detachment (as identified by the sudden fluorescence signal decay). At its maximum size the surface coverage was about 1200 µm^2^ on the valve tip. **c** Time-lapse images of blood aggregate building up over time on asymmetrical valves (see also Supplementary Movie 6). The aggregate forms slowly behind one of the leaflets. The arrow indicates the aggregate and the scale bar represents 100 µm. **d** The size of the aggregate becomes substantial after about 20 min and keeps growing exponentially. The continuous line is an exponential growth fit. The error bars represent standard error.

We then pulsed whole blood between 0 and 250 mbar, corresponding to the same flow rate used for the aqueous suspension of PS particles. We found that, for stiff valves (prepared with 50% PEGDA and 4% PI, corresponding to a Young’s modulus of about 67.8 ± 5.5 kPa), similarly to what discovered for PS particles, platelets accumulate preferentially at the valve tips where they form an aggregate. The adhesion of this clot to the valve tip is not as strong as the PS particles and as a consequence it detaches after reaching a critical size and forms again if pulsed flow is kept (Fig. 6 and Supplementary Movie 5). We then tested valves of lower stiffness (30% PEGDA and 4% PI, corresponding to a Young’s modulus of about 46.5 ± 5.2 kPa) again for the same pulsed flow conditions and found no accumulation anywhere on the valve or on the main channel. This confirmed that valve elasticity is crucial in modifying the flow pattern and to inhibit particles and platelets accumulation. Also, flexible valves react faster to the change in flow direction and can potentially detach the platelets that temporarily accumulate at their tips. These results imply that the mechanism of platelets accumulation is solely dependent on the flow conditions that develop due to a higher stiffness of the valve. In this study, we aimed to recapitulate primarily the interaction of the pulsatile type of flow, specific for veins, with flexible valve leaflets, and less emphasis was made to mimic the precise flow shear stress. In order to maintain hydrodynamic similarity (e.g., same Re) with different veins, the flow velocity in our microfluidic device has been adjusted proportionally. Hydrodynamic similarity implies that, despite the fact that the absolute value of the shear stress is different between our device and real veins, its role is proportionally the same in the fluid mechanics of the two systems. When it comes to the effect of shear on platelets agglomeration, it is the absolute value that counts. In the range of shear rates used in this study (<2500 s^−1^), platelet aggregation predominantly occurs via GPIIbIIIa interaction with fibrinogen, which requires activation of the integrin by soluble agonists that were absent in our experiments^42–44^. Aggregation through GPIb-von Willebrand factor (VWF) interactions, which does not require activation of GPIb, develops in much higher shear rates with full shift to this mechanism at shear rates above 10,000 s^−1^ ^45^. We did not observe any aggregates in the same shear conditions for a flexible valve. Altogether this suggests that platelet agglomeration found in our experiments is likely not induced by shear, and hydrodynamics is the main driving force behind the observed agglomeration. At the same time, interaction of VWF with GPIbalpha is implicated in DVT in the in vivo stenosis model^12^. This may be due to local release of highly adhesive ultra large multimers of VWF from Weibel–Palade bodies combined with limited supply of ADAMTS13, an enzyme that cleaves VWF, because of restricted blood flow.

Valve symmetry thus plays an important role in the formation of the flow patterns through the channel. Due to the laminar flow conditions combined with the identical valve’s mechanical characteristics, two symmetrical vortices develop, one after each valve leaflet (Supplementary Movies 1 and 3).

### Asymmetrical valve

It has been reported that leaflets of the same valve in a diseased vein may have different thickness/rigidity^39^. We thus fabricated valves with one leaflet stiffer than the other and studied how this affected particle accumulation. We noticed that in the asymmetric case there was a significant difference in the flow pattern and ultimately in the particle build up (Fig. 5).

In this condition, no vortex was formed after the valve as demonstrated by our GPV analyses and the time lapse videos recorded (Supplementary Movie 4). The asymmetry of the valve caused the more flexible leaflet (left in Fig. 5) to move within the pulsed flow whereas the stiffer leaflet (right in Fig. 5) remained almost stationary resulting in an asymmetrical flow pattern after the valve. This triggered particle accumulation in one of the valve pockets behind the leaflet, which proceeded faster compared to the symmetrical case (Supplementary Movie 4). When repeated with whole blood, the experiment showed a remarkable similarity with the results obtained for PS particles although the blood cells aggregate formed behind the valve was considerably smaller compared to the one made of PS particles and required a longer time to form (see Supplementary Movie 6).

Of note, in symmetrical conditions, both forward and back flows caused particles accumulation on the valve tips whereas in asymmetrical valves, back flow was the main cause for particle build up behind the valve. In both cases, increased stiffness enhanced or triggered particle accumulation, which is consistent with conditions leading to DVT.

In summary, we present herein an in vitro approach that combines in situ fabrication of flexible valves with tuneable elasticity and controlled flow in a microfluidic environment to produce prothrombotic conditions. By exploiting this novel approach, we were able to demonstrate how the stiffness of the valves influences the flow pattern in a vein and how this can be quantified by optical measurements. We then linked the flow behaviour to the presence of stagnant regions and vortices, and identified critical valve parameters, such as flexibility and flow rates, responsible for the formation of aggregates.

In particular, by using aqueous suspension of PS particles and whole blood, we showed for the first time that varying stiffness, and in particular stiffness asymmetry between valve leaflets, leads to different particle build up patterns. Independently of the sample observed, localised accumulation in symmetrical valves occurs on the valve tips and increases with increasing stiffness, whereas asymmetrical valves showed an accumulation in the valve pocket behind the leaflet. The asymmetry altered the flow patterns around the valve flaps forcing particles to get trapped in the recirculation zone behind the valve. Our system does not include other components of Virchow triad (blood coagulation and vascular wall alterations). This allows for more precise delineation of the specific role of flow geometry in thrombosis initiation but studies combining our model with other triad components should be the next step of this line of research.

Our results are consistent with findings that the risk of DVT increases with old age as valves become stiffer, and also suggest that circumstances where valve symmetries are not maintained might increase the risk of DVT.

Our work shows clear evidence that physical conditions, such as the characteristics of the flow or valves, are responsible for the flow behaviour in the veins, which is finally connected to different mechanisms of cell agglomeration. The entire complex of factors affecting flow geometry, some of which cannot be changed but can be tested in patients (e.g., flow velocity, vortices, or degree of valve leaflet flexibility), and others can also be modified (e.g., blood viscosity or haematocrit) may become a basis for more personalised prediction of DVT probability in the future. Modification of the latter group of factors may also be included in the complex of measures for DVT prevention.

Our in vitro model reflects the effect of physical parameters on the formation of thrombi happening in vivo. Thrombosis will be also influenced by biochemical aspects such as coagulation factors, nonetheless here we show how the physics of the venous system can represent a key that can potentially be exploited for prediction of DVT. As a notable example, our results highlighted how asymmetrical stiffness links to the formation of an aggregate behind the valve leaflet (where it is normally found in in vivo studies). This parameter has not been investigated so far and it could be a potential indication of a higher DVT risk.

## Methods

### Channel fabrication

Conventional photolithography techniques were used to fabricate microfluidic devices out of polydimethylsiloxane (PDMS) (Sylgard^®^ 184 Silicone Elastomer Kit, Dow Corning, UK)^25^. Briefly: SU8-2075 photoresist (MicroChem, Westborough, USA) was spin coated onto a silicon wafer (Si-Mat, Germany) and it was then exposed to ultra violet light using a mask aligner through a photo mask (Micro Lithography Services Ltd., UK). The uncured photoresist was then washed away to obtain a negative replica. PDMS (10:1 w/w ratio with curing agent) was then poured over the silicon wafer mould to create the microfluidic channel. The typical geometry consisted in a straight channel with dimensions: width, *W*, 300 μm, height, *H*, 80–100 μm and length, *L*, 2 cm. The PDMS was degassed under vacuum before curing it in an oven at 70 °C for 2 h. Once the PDMS was cured it was removed from the mould and used to fabricate the microfluidic device. Inlet and outlet were punched by a biopsy puncher (1.5 mm, Miltex by Kai) to create the top layer of the microfluidic device. For the bottom layer we used a glass slide spin coated with ≈250 μm of PDMS (30 s at 1500 rpm) and left to cure on a hot plate at 90 °C for 20 min. To assemble the device both layers were treated with a corona discharge (PZ2 Handheld Device, Relyon Plasma GmbH, Germany) and bonded together.

### Valve fabrication

The valves were made of polyethylene glycol diacrylate (PEGDA) (Mr ≈ 575, Sigma Aldrich, UK) and photo initiator (PI) 2-hydroxy-2-methyl propiophenone (Sigma Aldrich, UK). The PI permit the crosslinking of PEGDA when exposed to UV light with a wavelength, *λ*, of about 365 nm. First, a solution of PEGDA and PI was injected into the channel and left to settle ensuring that no flow was present within the device. A syringe was connected to the inlet and outlet to apply enough pressure to remove air bubbles as well as to stabilise the system. Once flow has settled a photo-mask with the desired geometry was placed at the conjugated plane in the epi-fluorescence light path of an optical microscope (Nikon Ti-U, Japan) and illuminated with an LED source containing a UV band (SOLA-SE-365, Lumencor, USA). To help with the alignment of the projection and channel and to prevent premature curing, a filter was used (*λ* = 545 ± 25 nm). The filter was then switched to allow the selection of the UV wavelength (*λ* = 350 ± 50 nm) to cure the PEGDA and reproduce the desired shape. This whole process was carried out using a 20× objective (Fig. 1a). PEGDA does not polymerise in the presence of oxygen^46^. As PDMS is oxygen-permeable, a thin layer of PEGDA (about 3–5 µm) remain liquid after UV exposure. This guarantee the possibility to fabricate valves that can move freely but also require to anchor them to keep them in place (Fig. 1a). The lowest exposure time required to achieve the best resolution was adopted, in our case 300 ms.

In order to create asymmetrical valves each leaflet was fabricate separately. We used a mask for each leaflet and controlled the characteristic of each one independently by modifying PEGDA or PI concentration or UV exposure time.

Finally, after valve fabrication, the channel was flushed with DI water and the whole device was submerged in water to preserve hydrophilicity until the experiment was performed, and in any case for no more than 24 h.

### In situ valve characterisation

In order to manipulate the elasticity of the valves fabricated by in situ photo-polymerisation, we altered their composition and exposure time to UV light. The mechanical properties are dependent on the concentrations of PEGDA, PI, and exposure time^46,47^.

**Fig. 7:**
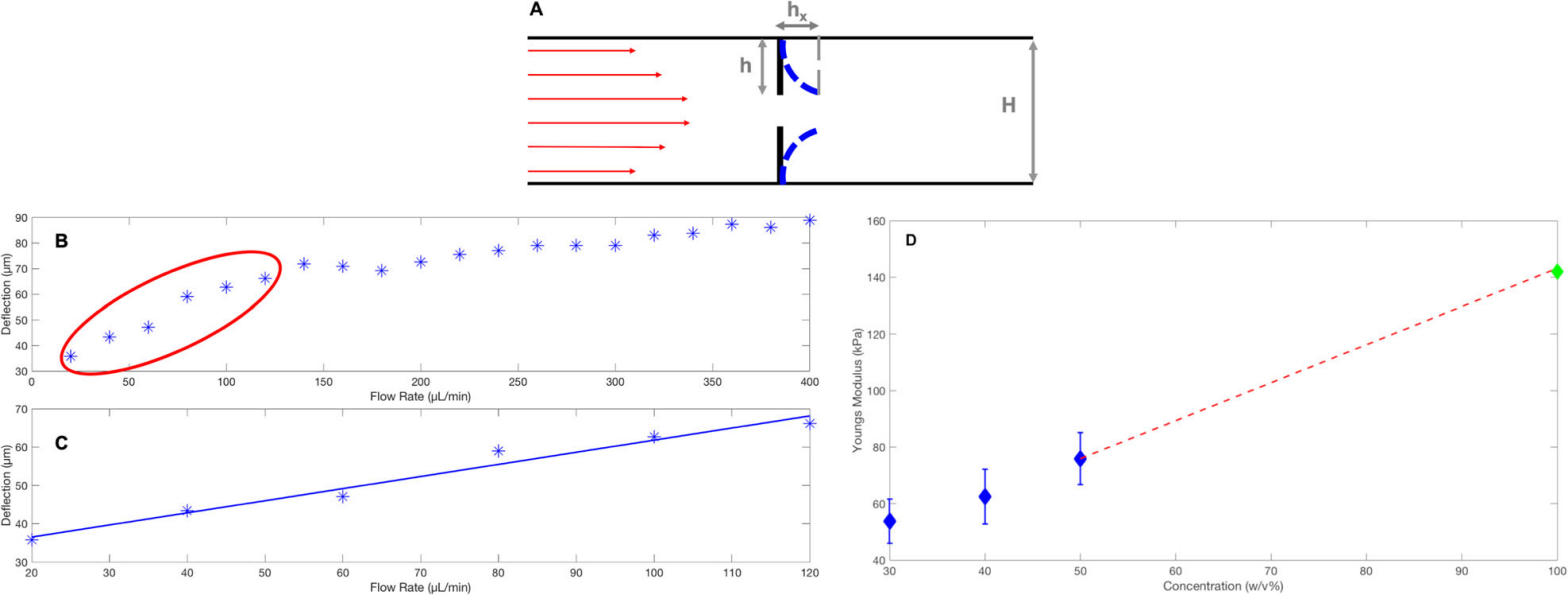
In situ Young’s modulus evaluation. **a** A schematic diagram of how the deflection, *h*_*x*_ is measured. **b** The deflections measured for different flow rates. **c** Zoomed in of the circled region in panel **b** designating the small linear deflection (first six points) which is used to calculate the Young’s modulus in plot D. Panel **b** and **c** refer to the case of 50% PEGDA concentration. The data shown in panel **d** refers to a fixed concentration of PI equal to 4% (w/v). The error bars represent standard error.

We characterised the elasticity of the valves by evaluating the Young’s modulus in situ as described previously^46,47^. It is in fact possible to link the deformation, *h*_*x*_, of the valve measured as seen in Fig. 7a, to the Young’s Modulus^46,47^. The results are shown in Fig. 7d where a linear extrapolation (red dashed line) to 100% (w/v) PEGDA gives the maximum reading of 142 kPa which is the maximum Young’s modulus for PEGDA 575 (one monomer chain between two cross-links) according to^47^

1 $$E = \frac{{3\rho RT}}{{M_w}},$$

where *E* is the Young’s modulus, *R* is the ideal gas constant, *T* is the temperature and *ρ* is the density.

### Ghost particle velocimetry

We tracked the flow exploiting GPV^26–29^. We used bright field microscopy and 200 nm PS particles (0.2% w/v, Sigma Aldrich, UK) as tracers. By reducing the numerical aperture of the condenser lens, we were able to detect the speckle pattern generated by the light scattered by the nanoparticles within the fluid using a fast camera (Photron, SA5). The speckle pattern is representative of the fluid motion as the PS particles are small enough to behave as passive tracers and the flow is considerably faster than the Brownian diffusion of the nanoparticles^26^. In addition, as the speckle pattern is uniform close to the interfaces, GPV permitted us to accurately map the flow field in the proximity of the flexible valve.

Enhancement of the speckle pattern is carried out using imageJ^38^ by subtracting the median of the total number of frames (more than 100) and thus removing the static contribution (see Supplementary Methods)^28^. It is then possible to track the speckle pattern using an open source MATLAB software, PIVlab^48^, that performs a cross correlation between each frames pair to obtain the flow displacement over time and permit to analyse the flow profiles including vortex detection and shear^48^. We captured the speckle pattern at various frames per second, fps, in the range 125–30,000 fps, adapting it to the flow rate and size of the device. This is done by allowing the average displacement of the flow to be less than half of the lateral size of the region of interest considered in the cross-correlation process.

To ensure accuracy of the GPV we compared the experimental results with the theoretical values expected as shown in the Supplementary Methods.

### Blood sample preparation

Whole blood was drawn from the cubital vein of three healthy donors for each series of experiments performed. The study conforms with principles outlined in the Declaration of Helsinki and informed consent was obtained from all donors. Blood was drawn from the cubital vein of healthy donors and stabilised with acid citrate dextrose in the ratio of 1:9. Platelet rich plasma was prepared by centrifugation at 200*g*, 20 min, room temperature (RT). Platelets were washed twice at 1000*g* in the presence of 10 µg PGI_2_ and resuspended in Tyrode’s–HEPES buffer (134 mM NaCl, 0.34 mM Na_2_HPO_4_, 2.9 mM KCl, 12 mM NaHCO_3_, 20 mM HEPES, 5 mM glucose, 1 mM MgCl_2_; pH 7.3). Washed platelets were allowed to rest for 30 min, were then labelled with calcein AM and returned to the whole blood, which was immediately used in microfluidics experiments.

### 3D printing

Larger moulds were printed using a resin 3D printer (Form 2 stereolithography 3D printer, Germany). The 3D printed channel mould was used to prepare a PDMS channel following the method mentioned previously (see channel fabrication). The valves were 3D printed separately and incorporated into the channel, before sealing the device with a glass slide.

**Fig. 8:**
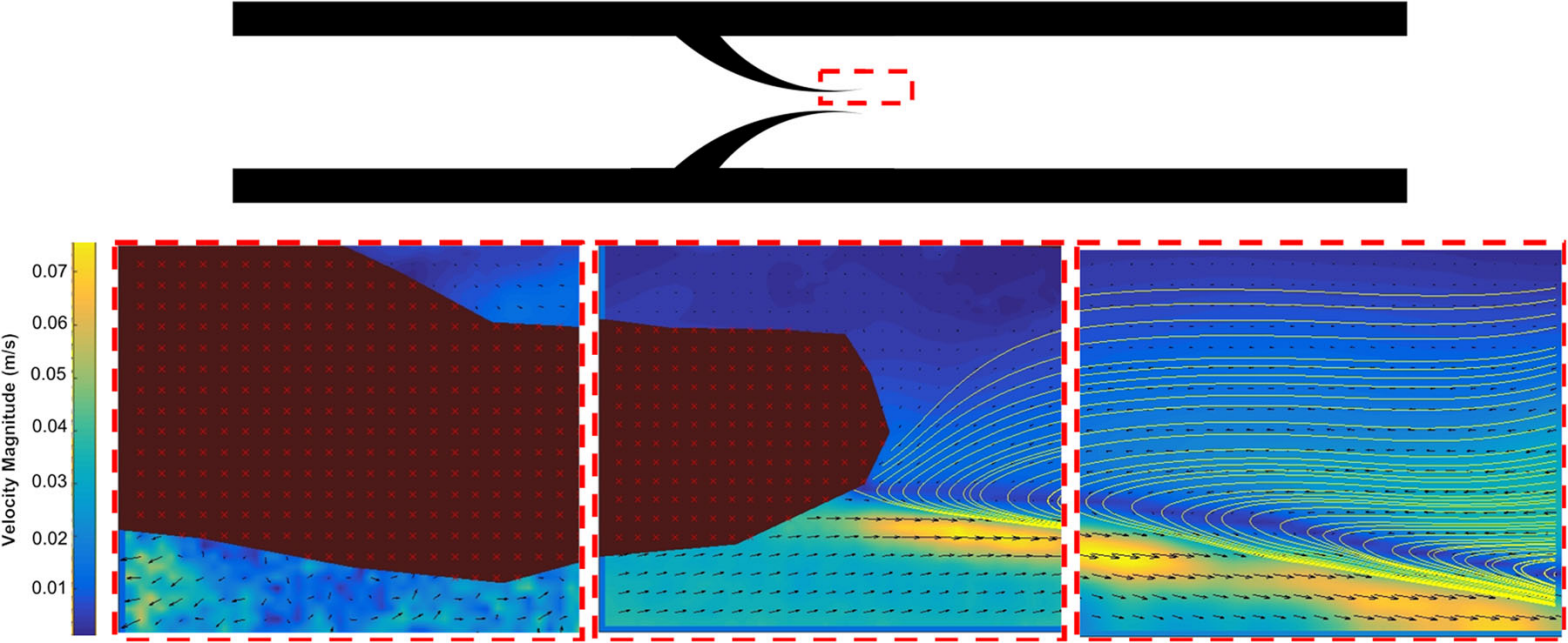
Vortex formation at Re = 163. A vortex forms at the valve tip (here only half vortex is shown as observed from multiple positions acquisition).

A 60 mL syringe was used to pump a 0.1% (w/v) 200 nm PS nanoparticle suspension through the large device for GPV detection of the flow patterns around the valve. A lower concentration of nanoparticles is needed to reduce the signal to noise ratio, as well as further reduction of the aperture on the condenser lens compared to the microfluidic model^29^. To increase the field of view a 10× magnification objective and acquisition in multiple positions along the axis of the channel was required (see Fig. 8).

## Supplementary information

Supplementary information

Description of Additional Supplementary Files

Supplementary Movie 1

Supplementary Movie 2

Supplementary Movie 3

Supplementary Movie 4

Supplementary Movie 5

Supplementary Movie 6

## Data Availability

The data that support the findings of this study are available from the corresponding author upon reasonable request.
