# Supplementary material for: The role of valve stiffness in the insurgence of deep vein thrombosis
Source: Commun Mater. 2020 Sep 16;1(1):65. doi: 10.1038/s43246-020-00066-2 (PMC7497694; doi:10.1038/s43246-020-00066-2)
Supplement: Supplementary file 1 — Supplementary information [file 43246_2020_66_MOESM1_ESM.pdf]

The role of valve stiffness on the insurgence of deep vein thrombosis

*Zoe Schofield [a,b], Hosam Alden Baksamawi [a], Joana Campos [c], Alessio Alexiadis [a], Gerard B. Nash [c], Alexander Brill [c,d,e, *], Daniele Vigolo [a,*]*

[a] School of Chemical Engineering, University of Birmingham, Birmingham, UK, B15 2TT
[b] Physical Sciences for Health, University of Birmingham, Birmingham, UK, B15 2TT
[c] Institute of Cardiovascular Sciences, University of Birmingham, Birmingham, UK, B15 2TT
[d] Department of Pathophysiology, Sechenov First Moscow State Medical University (Sechenov University), Moscow, Russia;
[e] Centre of Membrane Proteins and Receptors, University of Birmingham and Nottingham, The Midlands, United Kingdom

** Corresponding authors: d.vigolo@bham.ac.uk, A.Brill@bham.ac.uk*

Supplementary Methods

Analyses of Flow Profile by Ghost Particle Velocimetry

We analysed the flow pattern exploiting Ghost Particle Velocimetry (GPV), an optical technique that uses the speckle pattern generated by the white light scattered by sub-diffraction limit size particles to map the flow. We used an aqueous solution seeded with 200 nm polystyrene (PS) particles and we performed a cross correlation between pairs of consecutive frames via the Matlab software PIVlab[1]. We enhanced the signal to noise ratio by subtracting the median, obtained from at least 100 frames, from each frame as described in a previous work[2]. In this way, as evidenced by Supplementary Figure 1, it is possible to remove the static contribution and make the speckle pattern clearly visible.

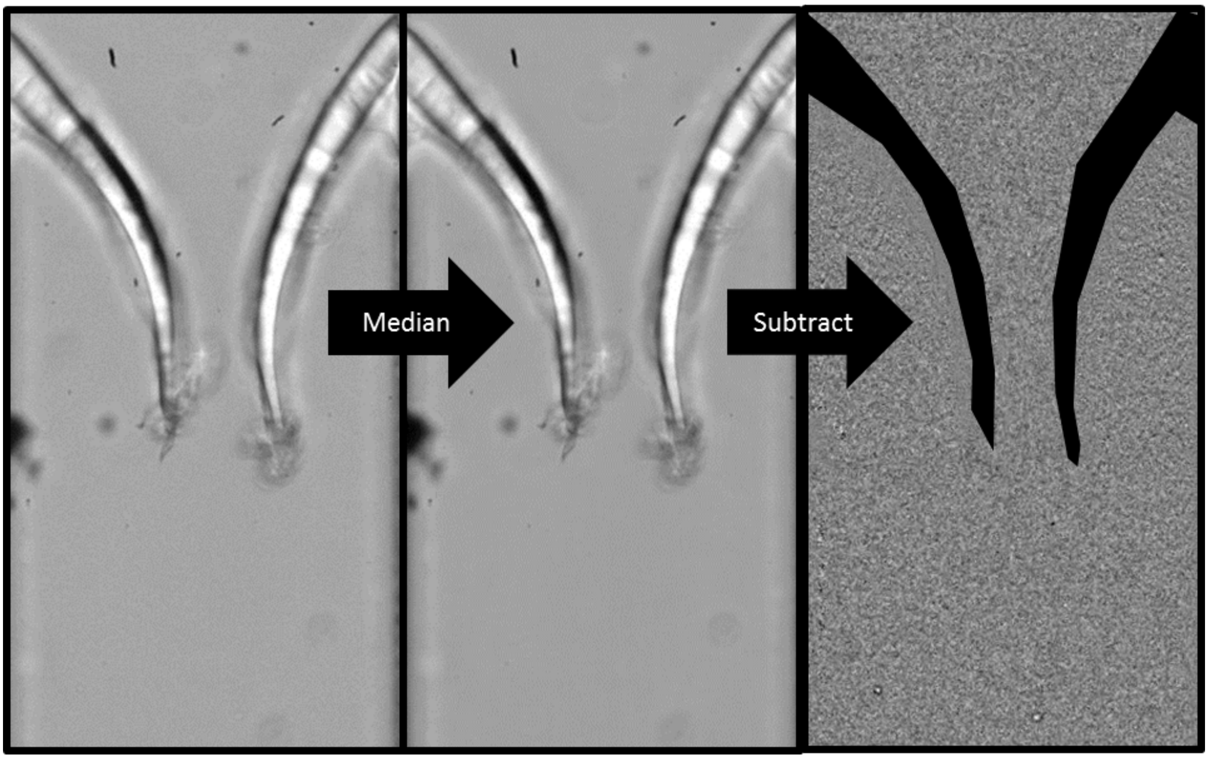

Supplementary Figure 1: A visual representation of the image processing carried out. From the left, the figure shows the original bright field image followed by the median image of 100 frames (the static contribution). When the median is subtracted from each frame a speckle pattern remains (figure on the right). We masked in black the valve to make it visible after median subtraction.

In order to confirm the validity of GPV to determine the flow pattern and quantify the flow velocity in our geometry, we performed a control experiment in which we evaluated the velocity in the unconstrained channel, before the valve. We compared the measurement obtained from the analyses of the GPV images to the theoretical mean flow velocity expected in the same cross section for the given flow rate so that $v = Q/A$, where v is the fluid velocity, Q the flow rate and A the cross section. An example is shown in Supplementary Figure 2. It is worth noticing that the experimental values are slightly higher than the theoretical. This is however expected considering that the measurements are taken in the middle plane where the average velocity is maximal and only averaged over a volume of about 20-30 µm of thickness rather than the overall volume. This is due to the inherent capability of GPV to extract information from a layer of thickness δ_z in the direction perpendicular to the field of view proportional to the numerical aperture of the condenser lens used, NA_C, following $\delta_z \approx \lambda/(NA_C)^2$. In our setup $NA_C \approx 0.15 - 0.2$, and thus $\delta_z \approx 20 - 30\ \mu m$.

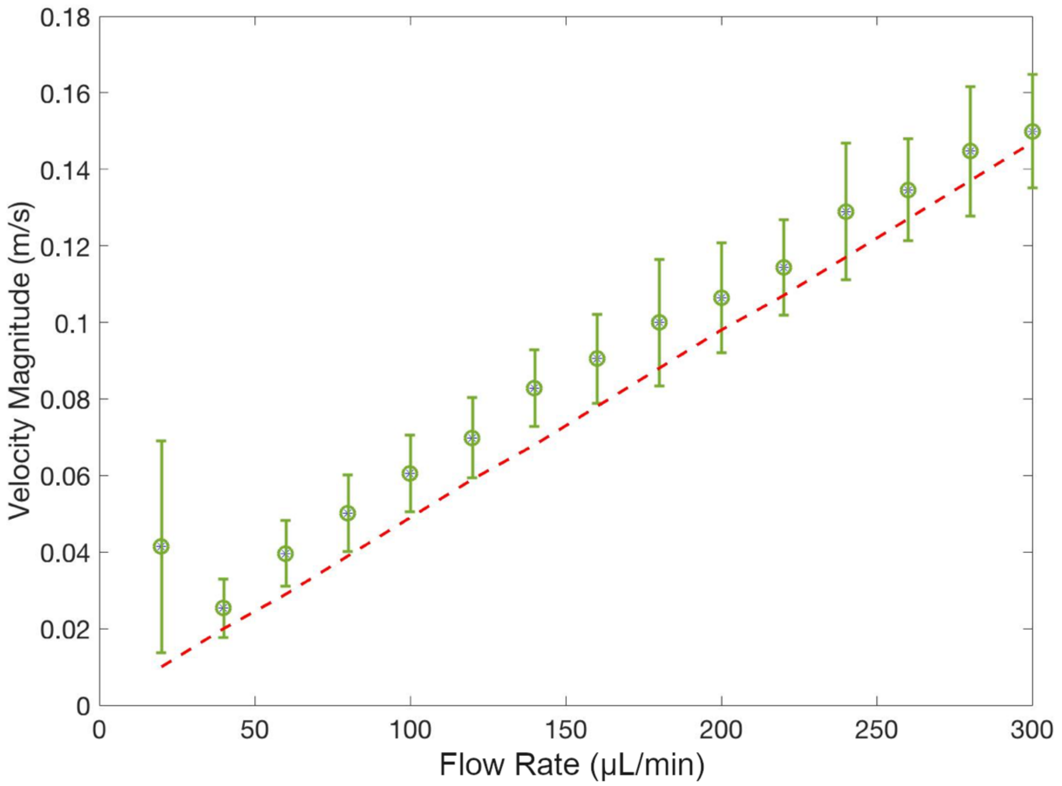

Supplementary Figure 2: Graph illustrating the velocity magnitude comparison with the theoretical values. The data points are the mean average over multiple GPV experiments with error bars indicating the standard error. The dashed line corresponds to the theoretical value of the mean velocity.

Velocity Profiles Across and Behind the Valve

When investigating the velocity profiles in the vicinity of the valve, there are two regions with very different average velocities. Across the valve the flow forms a jet with an average velocity higher of the one measured in the main channel. Once the valve opens it also creates two pockets behind the leaflets. Here the flow is almost stagnant. Further down after the valve a pair of vortices develops at the tips of the valve. In order to collect data from the whole valve area we acquire high speed videos at different frame rates. Supplementary Figure 3 shows how the choice of different frame rates affected the results we collected. It is important to note how the most relevant flow pattern for this study is the one that develop at low speed behind the valve and immediately after rather than the jet between the leaflet. This is the reason why we focused our results in these areas preferring to acquire videos at a frame rate adequate to map the vortices and the recirculation happening in the stagnant regions in the valve pockets. The frame rate was thus reduced to permit the evaluation of slow flow velocities that cannot be mapped at high frame rate due to the displacement of the flow from frame to frame being too minute. As a result, the regions with high velocity cannot be mapped at these acquisition rates.

Moreover, increasing the frame rate implies an increase of the signal to noise ratio and a reduced resolution (the fast camera used requires a reduced resolution for high frame rates). As a result, we were able to accurately capture flow velocities of up to 0.2 m/s by capturing video at 30,000 frames per second.

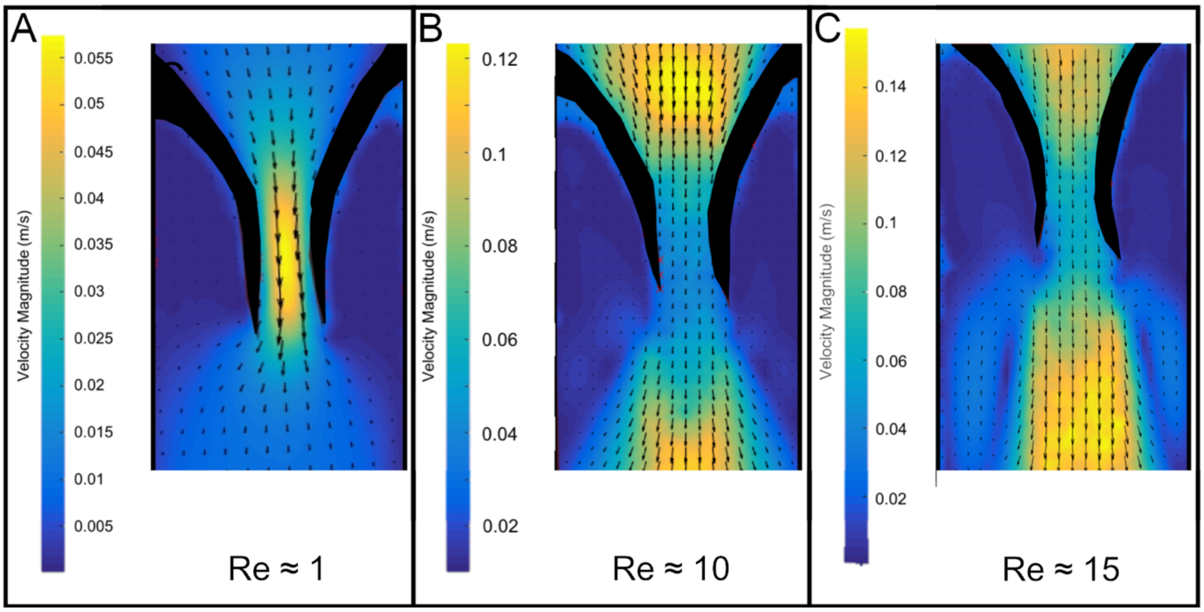

Supplementary Figure 3: Flow map at the valve at different frame rates for different Reynolds numbers (Re). A – at low Re it is possible to accurately map at the same time both the stagnant regions and the jet between the valve leaflets. B and C – at increasingly higher Re we used a frame rate that permit an accurate evaluation of the velocity only for the stagnant regions. As a consequence, the jet region resulted in erroneous evaluation of the velocity that appears lower than the one before (or immediately after) the valve.

Characterisation of vortex size and circulation at different Reynolds numbers

It is possible to link the vortex area with the circulation at different Re.

The vortex area is calculated as the area of the vortex corresponding to the maximum circulation. We consistently followed the procedure accompanying PIVlab[1,3], the MATLAB based software that we used to perform the GPV analyses. Briefly, the circulation increases with the radius of the vortex (from the centre outwards), reaching a plateau at its maximum value at the edge of the vortex. The vortex area we considered corresponded to the minimum vortex radius to achieve maximum circulation.

In addition to what already shown in the main text, it is interesting to note how the vortex area has a linear dependence on the circulation so that for increasing tangential velocity of the vortices, the surface covered by each vortex is also proportionally larger as shown in Supplementary Figure 4 where we plot the average circulation values versus the vortex area, evidencing maximal and minimal circulation. The vortices kept elongating more and more along the channel with no sign of plateauing, signifying that the geometrical constrained imposed by the channel does not affected the size of the vortices that eventually became more anisotropic and developed in length as width and depth were limited by the physical size of the microchannel. Unfortunately, we were not able to analyse the vortex size for Re > 34 due to experimental limitations as it would have been interesting to investigate the evolution of the vortex size for faster flow rates.

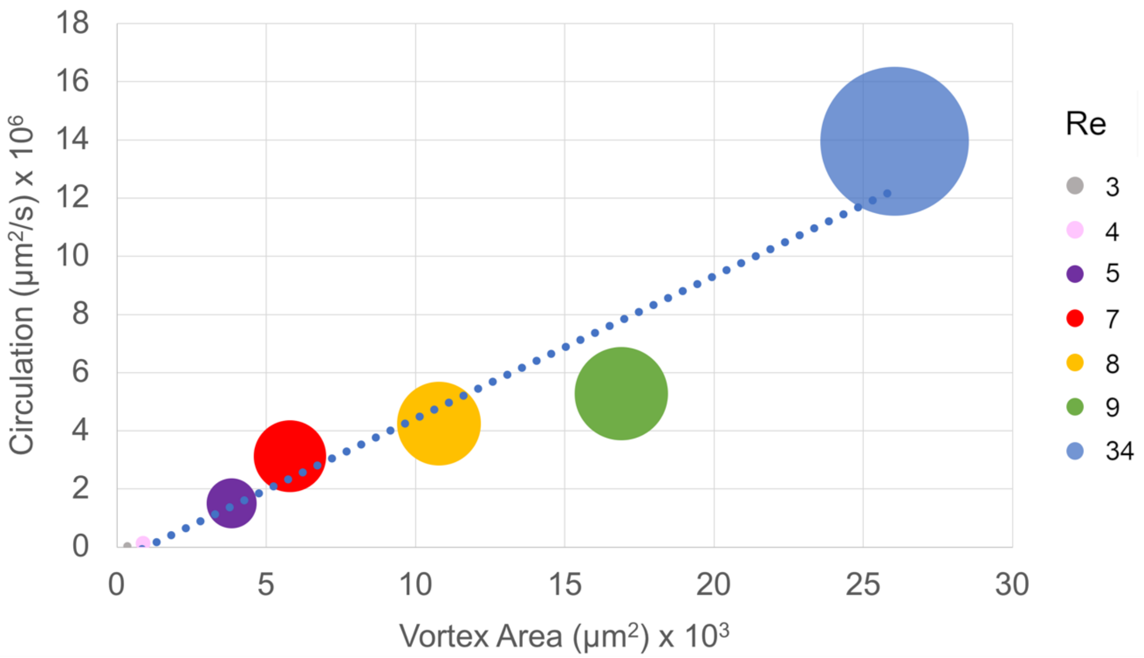

Supplementary Figure 4: Circulation correlates with vortex area. For each Re the size of the marker point represents the minimum and maximum circulation calculated for the vortex developing at the tip of the valve. The dashed line represents a linear fit ($y = 0.49x - 0.48$; $R^2 = 0.9265$).

Accumulation of Particles

Particle accumulation is influenced by the flow velocity. In Supplementary Figure 5, it is evident that at a higher flow rate (150 µL min^{-1}) there is less accumulation between the valves in comparison to the lower flow rate (50 µL min^{-1}).

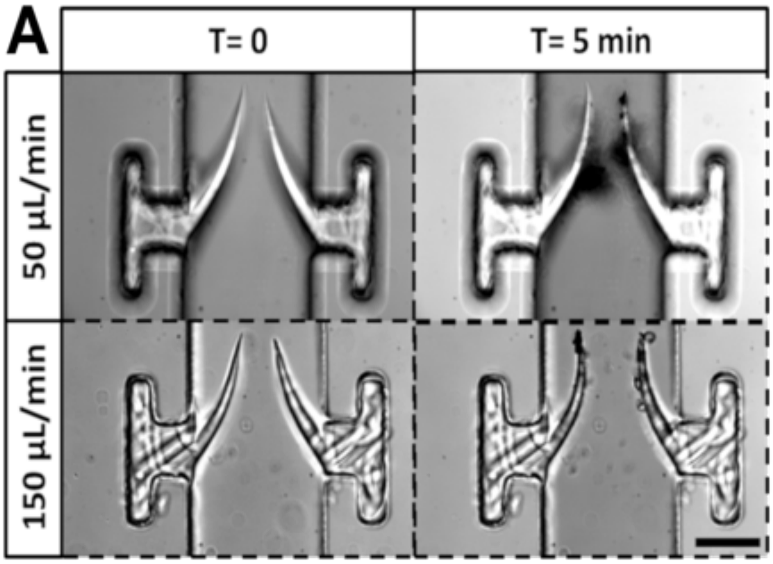

Supplementary Figure 5: – Particle accumulation for different flow rates in the case of symmetrical valve rigidity and continuous flow condition. The scale bar corresponds to 150 µm.

Supplementary References

1. Thielicke, W. & Stamhuis, E. J. PIVlab – Towards User-friendly, Affordable and Accurate Digital Particle Image Velocimetry in MATLAB. *J. Open Res. Softw.* **2**, (2014).
2. Pirbodaghi, T., Vigolo, D., Akbari, S. & DeMello, A. Investigating the fluid dynamics of rapid processes within microfluidic devices using bright-field microscopy. *Lab Chip* **15**, 2140–2144 (2015).
3. Thielicke, W. PIVlab tutorial. Available at: https://pivlab.blogspot.com/p/blog-page_19.html. (Accessed: 24th February 2020)
