# Supplementary material for: The role of valve stiffness in the insurgence of deep vein thrombosis
Source: Commun Mater. 2020 Sep 16;1(1):65. doi: 10.1038/s43246-020-00066-2 (PMC7497694; doi:10.1038/s43246-020-00066-2)
Supplement: Supplementary file 2 — Description of Additional Supplementary Files [file 43246_2020_66_MOESM2_ESM.pdf]

Description of Additional Supplementary Files

File Name: Supplementary Movie 1
Description: GPV data of the flow through a valve where a pair of vortices develop due to sufficiently high flow rate and sufficiently high stiffness of the valve. This Movie refers to the case of a valve fabricated with [PEGDA] = 50% and [PI] = 8% and a flow rate of 120 μL/min.

File Name: Supplementary Movie 2
Description: GPV data of the flow through a valve where no vortex develops due to low flow rate and/or low stiffness of the valve. This Movie refers to the case of a valve fabricated with [PEGDA] = 50% and [PI] = 8% and a flow rate of 20 μL/min.

File Name: Supplementary Movie 3
Description: 10 μm PS particles flowing through a valve while pulsating the flow at 1 Hz between 0 and 100 mbar. In this video the valve has leaflets with identical stiffness (i.e., symmetrical) and the particle accumulation happens at the valve tip. Moreover, it is easy to observe a pair of vortices forming right after the valve.

File Name: Supplementary Movie 4
Description: 10 μm PS particles flowing through a valve while pulsating the flow at 1 Hz between 0 and 100 mbar. In this video the valve has leaflets with different stiffness (i.e., asymmetrical). In particular, the left leaflet is softer than the leaflet on the right and the particle accumulation happens behind the valve. In this case no vortex develops due to the asymmetry in the flow pattern generated by the asymmetrical behaviour of the valve leaflets.

File Name: Supplementary Movie 5
Description: accumulation of fluorescently labelled platelets in whole blood flowing within the microfluidic device with valves presenting identical stiffness (i.e., symmetric). The flow is pulsed at 1 Hz between 0 and 300 mbar. The video shows the relatively fast accumulation and subsequent detachment happening at the tip of the valve.

File Name: Supplementary Movie 6
Description: accumulation of fluorescently labelled platelets in whole blood flowing within the microfluidic device with valves presenting different stiffness (i.e., asymmetric). In particular, the upper leaflet is more flexible than the lower one. The flow is pulsed at 1 Hz between 0 and 300 mbar. The video shows the exponential build up behind a leaflet due to the asymmetric flow pattern.
